# Abstract of the Proceedings of the Buffalo Medical Association August 3d and September 7th

**Published:** 1875-09

**Authors:** E. N. Brush

**Affiliations:** Secretary


ART. II.—*Abstract of the Proceedings of the Buffalo Medical Association.* Aug. 3d and Sept. 7th. Reported by E. N. Brush, M. D., Secretary.

The President, Dr. Gould in the chair.

Members present—Drs. Gould, White. Fowler, Howe, Boysen, Brecht, Brush, Hebenstreit, and by invitation Dr. Shannon, U. S. A.

The minutes of the previous meeting were read and approved. The application of Dr. M. B. Folwell for membership was received and under the rules laid upon the table for one month.

The application of Dr. Herman Mynter was taken from the table and he was unanimously elected a member.

Dr. Lucien Howe presented the eye of a man, who, on the 3d day of July received a wound on the cornea of the left eye extending into the sclerotic for one fourth of an inch. The wound opened into and evaporated the anterior chamber. When first seen, on the tenth, the lens was pressed into the wound pushing the iris before it. From the tenth until the thirteenth the patient did well; the wound in the sclerotic was healed, and that in the cornea nearly so. From this time, however, pain and tenderness began to manifest themselves, and on the twenty-ninth the condition was so bad that enucleation was found necessary in order to preserve the uninjured eye. The case was a good illustration of the danger attending wounds in the cilliary region. In the specimen presented the cornea was infiltrated with pus. Over the anterior portion of the iris a deposit had formed of a serous character. The lens had become broken up and absorbed. The choroid and sclerotic were inflated with blood, the vitreous filled with a flocculent deposit. The stump, in this case, healed nicely, and on the ninth day after the operation the man resumed his work.

Dr. White related a case of atresia of the vagina. He remarked that atresia was not an unfrequent occurrence, several cases having fallen under his observation from imperforate hymen and other causes; but occlusion following, and the result of labor, was of rare occurrence.

The patient was confined in 1873, considerable *post partum* hemorrhage occurring, a cotton plug was pushed into the vagina, saturated with acetic acid. Convalescence was tardy. At the end of several months a menstrual molimen appeared, but was not accompanied by any flow. Several physicians saw the case, but the cause was not suspected. At last, upon making a vaginal examination, Dr. Griffin, of North East, discovered the occlusion and made an effort in which he partially succeeded, separating the vulva, but finding complete occlusion of the vagina at its middle. When Dr. White first examined the patient, he found the vagina firmly closed by what appeared to be a firmly organized diaphragm. A finger introduced into the rectum could detect a tumor above the obstruction, which presented some obstruction to defecation. The tumor was thoroughly distended and evidently contained fluid.

The patient having been placed under the influence of Ether by Dr. Rochester, Dr. White proceeded to open up the passage, the operation being made with as little cutting as possible. As soon as an opening was made about three pints of thick, tarry substance escaped, the retained menstrual fluid. A dilator was introduced and in three weeks the patient returned to her home well.

This case was mentioned on account of its rarity. Whether the adhesion of the parts was due to the application of the acetic acid or to the long continued pressure of the head could not be stated. He had heretofore related in this association two or three similar cases. One in which the occlusion was relieved seven years after its production. In this case the os uteri was closed. In another case a patient of Dr. Potter, of Attica, after breaking down the occluding bands, the uterus was found in a condition of atrophy, it having become reduced to almost infantile size. The canal at the neck was almost closed and the neck so shortened that it could scarcely be distinguished. In the presence of Drs. Coventry, Moore and Eastman the uterine canal was incised and then dilated, and a galvanic pessary introduced to excite its function. In a few years the patient gave birth to a boy, thus illustrating the complete restoration of the uterine function. Dr. White also reported that he had been recently making pretty free use of the Squibb's solid extract of ergot, hypodermically, and also of a German preparation of Ergotine, in uterine fibroids. Of course pediculated intra-uterine fibroids could be removed by other means, but in intra-mural and sub-mucous fibroids the employment of ergot, as indicated, would produce uterine contractions and thus arrest the growth, if not cause its entire absorption. The use of ergot in sub-peritoneal or extra-uterine fibroids he did not think would be attended by any benefit. At the meeting of the American Medical Association in Louisville, Dr. Byford read a paper on this subject, and reported several successful cases. Dr. White had also used Ergotine in these cases by suppository, passing into the rectum five to ten grains every night, the solid extract and also the ergotine being employed. In a patient now under treatment it had been tried in several ways, but the administration, per rectum, was the only method in which it could be tolerated. He gave preference to the Squibb's solid extract.

On motion the Association adjourned.

Sept. 7th.—The President, Dr. Gould, in the Chair. Present—Drs. White, Rochester, J. F. Miner, W. W. Miner, Samo, Bartlett, Brecht, Fowler, Strong, Howe, Abbott, Mynter, L. F. Harvey, Hopkins, Brush and Wetmore, and by invitation, Dr. Shannon, U. S. A.

The minutes of the previous meeting were read and approved.

The application of Dr. L. H. Long, for membership was received and under the rules was laid upon the table for one month. The application of Dr. M. B. Folwell was taken from the table and he was unanimously elected a member.

The Secretary reported the Constitution and By-Laws printed and ready for distribution.

Dr. F. W. Abbott read an essay upon the *Cornea in Health and Disease* (see page 41). At the conclusion of the essay he presented three patients, who illustrated points taken in his paper. The first was the case of tattooing, described in the paper. The color of the opacity was seen to be wholly obliterated by the operation. The other two were illustrations of ulcer of the cornea, almost wholly healed without opacity, and of extensive lucoma.

Dr. Howe moved that the thanks of the Association be extended to Dr. Abbott.

Dr. White said that in arising to second the motion, he wished to make a few remarks, which, though foreign to the subject under consideration, were suggested by the paper. He had reference to the pursuit of special departments of practice by members of the medical profession. While he was heartily in favor of special work by those who were by study and observation adapted to its pursuit, he wished to make the assertion that the ground work of special practice should be laid in the broad field of general medical education and practice, that no man could be a good specialist who had not first been a general practitioner. There was but one practical point in the paper to which he wished to refer, that was concerning the application of nitrate of silver. It had been a professional life-long practice with him to use it in diseases of the conjunctiva and cornea. From this paper he had learned that it is not only desirable to heal the ulcer, but it is desirable to do so in a particular manner. He had not given much attention to this subject, having been in the habit, when possible, of referring this class of patients to others. The large attendance at the meeting illustrated the value of having designated essays or subjects for discussion, as it added interest to the meetings and drew out members. The younger members should not hesitate to present papers for discussion or comment

The motion of Dr. Howe was carried.

Dr. Bartlett asked if any benefit was derived from counter-irritation.

Dr. Abbott replied that in recurrent phlyctenulæ, authors recommended the seton or blister, but he had not been in the habit of using them and could not speak from his own observation.

Dr. Strong said that early in his practice he had used, with benefit, nitrate of silver, applied to the lids externally and would ask if it was now employed.

Dr. Howe remarked that Bowman and Crichett, and also Lawson employed counter-irritation in the form of setons and blisters with benefit in intractable cases.

In tattooing, which was attracting considerable attention, the great objection was the want of a proper instrument. In one case which came under his notice, a good operator in Guy's Hospital, punctured the cornea for this reason.

Dr. Miner said he had a theoretical, but no practical knowledge of tattooing, but from his knowledge of surgical procedures, he was of the opinion that the more simple the instrument employed in the operation the better, providing it be used with care. He could see no reason why India Ink could not be used to color the new tissue constituting opacity of the cornea as readily as it could be used in making images upon the arm of the sailor. He hoped the instrument used would be a simple needle so that the point could be constantly under the eye of the operator. As to counter-irritation in inflammatory diseases of the globe of the eye, he could see no good reason why such practice should be any longer continued. Thirty years ago it was his opportunity to attend the New York Eye Infirmary. Cupping and leeching were then in fashion. It was customary to arrange twenty or thirty poor, half fed, half clothed victims in a row, and two days in a week apply cups to the temples. The dark room, torch light of a wick dipped in alcohol and lighted, the slight of hand operation by which a spring lancet and a cup were applied to the temple, sometimes on both sides, forcibly reminded one of Satan, superintending the Infernal Regions. It was there observed that the same patients appeared from week to week. He was happy to say the practice had long since been abandoned. Not only in the New York Infirmary, but everywhere, in good treatment of the inflammatory affections having their seat mainly on the conjunctival covering of the eye.

Dr. Bartlett said that while he had no doubt but that Dr. Miner was strong in his convictions in regard to counter-irritation, he had noticed cases in which the improvement could be attributed to the application of a blister back of the ear or on the temple.

Dr. Abbott said that he had neglected to speak of the necessity of administering chloroform in some cases to children, in order to get a perfect examination of the eye. He had noticed, in these cases, that after the administration of the anæsthetic, the photophobia was sometimes less intense.

Dr. Strong moved that Dr. Bartlett be invited to read an essay at the next meeting on Ozone. Seconded by Dr. Hopkins, and carried.

On motion the Association adjourned.

